# A species-independent lateral flow microarray immunoassay to detect WNV and USUV NS1-specific antibodies in serum

**DOI:** 10.1016/j.onehlt.2023.100668

**Published:** 2023-12-27

**Authors:** Bijan Godarzi, Felicity Chandler, Anne van der Linden, Reina S. Sikkema, Erwin de Bruin, Edwin Veldhuizen, Aart van Amerongen, Andrea Gröne

**Affiliations:** a Department of Biomolecular Health Sciences, Utrecht University, Yalelaan 1, 3584 CL Utrecht, the Netherlands; b BioSensing & Diagnostics, Wageningen University and Research, Bornse Weilanden 9, 6708 WG Wageningen, the Netherlands; c Department of Viroscience, Erasmus MC, Wytemaweg 80, 3015CN Rotterdam, the Netherlands

**Keywords:** Arboviruses, West Nile Virus, Usutu Virus, One Health, Lateral Flow Assay (LFA), Serology

## Abstract

Arboviruses such as West Nile Virus (WNV) and Usutu Virus (USUV) are emerging pathogens that circulate between mosquitoes and birds, occasionally spilling over into humans and horses. Current serological screening methods require access to a well-equipped laboratory and are not currently available for on-site analysis. As a proof of concept, we propose here a species-independent lateral flow microarray immunoassay (LMIA) able to quickly detect and distinguish between WNV Non-Structural 1 (NS1) and USUV NS1-specific antibodies. A double antigen approach was used to test sera collected from humans, horses, European jackdaws (*Corvus monedula)*, and common blackbirds (*Turdus merula)*. Optimization of the concentration of capture antigen spotted on the LMIA membrane and the amount of detection antigen conjugated to detector particles indicated that maximizing both parameters increased assay sensitivity. Upon screening of a larger serum panel, the optimized LMIA showed significantly higher spot intensity for a homologous binding event. Using a Receiver Operating Characteristics (ROC) curve, WNV NS1 LMIA results in humans, horses, and *C. monedula* showed good correlation when compared to “gold standard” WNV FRNT90. The most optimal derived sensitivity and specificity of the WNV NS1 LMIA relative to corresponding WNV FRNT90-confirmed sera were determined to be 96% and 86%, respectively. While further optimization is required, this study demonstrates the feasibility of developing a species-independent LMIA for on-site analysis of WNV, USUV, and other arboviruses. Such a tool would be useful for the on-site screening and monitoring of relevant species in more remote or low-income regions.

## Highlights

• In this study, a species-independent lateral flow microarray immunoassay (LMIA) was developed and tested.
• LMIA capture and detection antigen concentrations were optimized prior to testing of larger serum panel.
• The LMIA distinguished between WNV NS1 and USUV NS1-specific antibodies in sera of four species.
• For the four species tested, LMIA results were comparable to FRNT90-confirmed results.

## 1 Introduction

Arboviruses, viruses that are spread by arthropods such as mosquitoes and ticks, are considered emerging pathogens due their incidence rate continuing to increase in previously unaffected regions [1]. The impact of factors such as climate change and globalization may influence the further dissemination of arboviruses into previously unaffected regions, and it is therefore important to survey these regions for the introduction and dissemination of these arboviruses [2].

West Nile Virus (WNV) and Usutu Virus (USUV) are arboviruses in the *Flaviviridae* family that circulate between various mosquito and bird species within enzootic cycles, occasionally spilling over into dead-end hosts including humans and horses [[3], [4], [5]]. WNV and USUV were first detected in Europe in 1962 and 2001, respectively, and both have since spread to many other European countries [1,6,7]. In the Netherlands, WNV was first detected in common whitethroat in 2020 and subsequently detected in humans in the same year [8,9]. Similarly, USUV was detected in birds in 2016 and has since also been detected in Dutch blood donors [10,11]. Direct detection of WNV and USUV can be challenging due to a short viraemic phase. It is because of comparatively longer detection windows that serological methods are therefore used to detect arbovirus-specific antibodies and play a crucial role in surveillance [[12], [13], [14]].

Amongst current serological methods used to detect arboviruses-specific antibodies, enzyme linked immunosorbent assays (ELISA) and neutralization tests (NT) are the most common. While ELISAs are relatively rapid and inexpensive, they are generally less specific, and results must be confirmed by a NT such as focus reduction neutralization test (FRNT) [12]. This is particularly important regarding WNV and USUV as both have been shown to co-circulate in parts of Europe and exhibit a high degree of serological cross-reactivity [1].

The high degree of serological cross-reactivity is attributable to WNV and USUV belonging to the same Japanese Encephalitis Virus (JEV) serocomplex, meaning their envelope (E) antigen as well as their non-structural 1 (NS1) antigen share a high amino acid homology. One proposed method to address this issue is to determine if there is a four-fold or greater difference in one or more quantitative serological methods [15]. However, achieving a four-fold serological differentiation between WNV and USUV can still be difficult [3,13,16]. So, while ELISAs are relatively rapid and inexpensive, they are not highly specific. Meanwhile, NTs are highly specific but are time-consuming and require a BSL 2 or 3 facility. For both methods, access to a well-equipped laboratory may not be problematic in wealthier countries, but this can be problematic in lower income and more remote regions.

Lateral flow assays (LFAs) are a well-established method to rapidly analyse samples on-site and do not require a well-equipped laboratory to run. Thus, LFAs have a great advantage in the field or in circumstances without access to well-equipped laboratories. To determine the feasibility of developing a species-independent LFA able to detect and distinguish between WNV NS1 and USUV NS1-specific antibodies, a lateral flow microarray immunoassay (LMIA) utilizing a double antigen approach was developed as a proof of concept. Since there is no species-specific antibody used as either capture or detection antigen, the double antigen approach allows the assay to be species independent. Fitting into a One Health approach, the usefulness of a species-independent serological assay is evident. For example, various avian species act as reservoir hosts for WNV while spill over events can affect dead-end hosts such as humans and horses. To analyse the performance of the proposed LMIA, FRNT90-confirmed sera from multiple species were tested. If successful, the proposed LMIA would be a useful on-site screening tool for the surveillance of WNV and USUV.

## 2 Materials and methods

### 2.1 Serum samples tested

See Table 1.

**Table 1:** Summary of panel of sera used in this study.

Species	Virus or vaccine	Infection mode	# serum samples	# specimen	DPI/DPV range	Confirmatory test	Origin	Ref.
Horse	WNV	Exp.	11	11	Unknown	PMA and FRNT90	NL	
		Exp.	4	1	0–21	PMA and FRNT90	NL	
	Negative		1	Pool of 20		ELISA	NL	
Human	WNV	Natural	3	2	0, 7	PMA and FRNT90	Romania (2017/19)	[17]
		Natural	2	2	0–7	PMA and FRNT90	Bosnia-Herzegovina (2018/19)	[17]
		Natural	5	2	0–60	PMA and FRNT90	Croatia (2018)	[17]
	Negative		1	1		PMA and FRNT90	NL (2022)	
European jackdaw (*Corvus monedula)*	WNV	Exp.	8	8	8	PMA and FRNT90	NL	[18]
Common blackbird (*Turdus merula)*	USUV	Natural	9	9	Unknown	PMA and FRNT90	NL (2016)	[8,10]
Total			44					

Abbreviations: Exp. = Experimental; DPI = Days post infection; DPV = Days post vaccination; PMA = Protein microarray; NL = the Netherlands.

### 2.2 Antigen production

Commercially available WNV (strain NY99, Uniprot Q9Q6P4) and USUV ( strain Vienna 2001, NCBI Accession Number: AWC68492.1) recombinant NS1 antigen were produced in HEK-293 cells (The Native Antigen Company, Oxfordshire, UK).  .

### 2.3 Conjugation of antigen

Detection antigen conjugates were prepared as previously described with modifications [19]. Prior to conjugation, WNV NS1 and USUV NS1 antigens were desalted using Amicon® Ultra-0.5 Centrifugal Filter Devices (Merck, Darmstadt, Germany). Desalted protein concentration was determined using a spectrophotometer (DeNovix DS-11 FX, Wilmington, USA). Multiple detection antigen conjugates were prepared; firstly, 1 mL of 0.2% sonicated carbon nanoparticle suspension (borate buffer (BB) (5 mM, pH 8.8)) was mixed with 350, 175, or 88 μg of WNV NS1 such that final concentration was 350, 175, or 88 μg WNV NS1/mL 0.2% carbon nanoparticle suspension (CNP) suspension. Secondly, 1 mL of 0.2% sonicated CNP suspension was mixed with 350, 175, or 88 μg of USUV NS1 such that final concentration was 350, 175, or 88 μg USUV NS1/mL 0.2% CNP suspension. Finally, 1 mL of 0.2% sonicated CNP suspension was mixed with 350 μg/mL of BSA-DNP such that final concentration was 350 μg BSA-DNP/mL 0.2% CNP suspension. These preparations were stirred overnight at 4 °C  on a magnetic stirrer and subsequently centrifuged at 13636 ×*g* (4 °C, 15 min, Sigma 2 K15, Sigma Laborzentrifugen GmbH, Osterode am Harz, Germany). Each set of preparations was washed twice in wash buffer ( BB, 5 mM, pH 8.8 containing 1% skim milk protein) and resuspended to 0.2% in storage buffer (BB, 100 mM, pH 8.8 containing 1% skim milk protein). The prepared conjugates were stored at 4 °C until further use.

### 2.4 Preparation of LMIAs for optimization

**Fig. 1:**
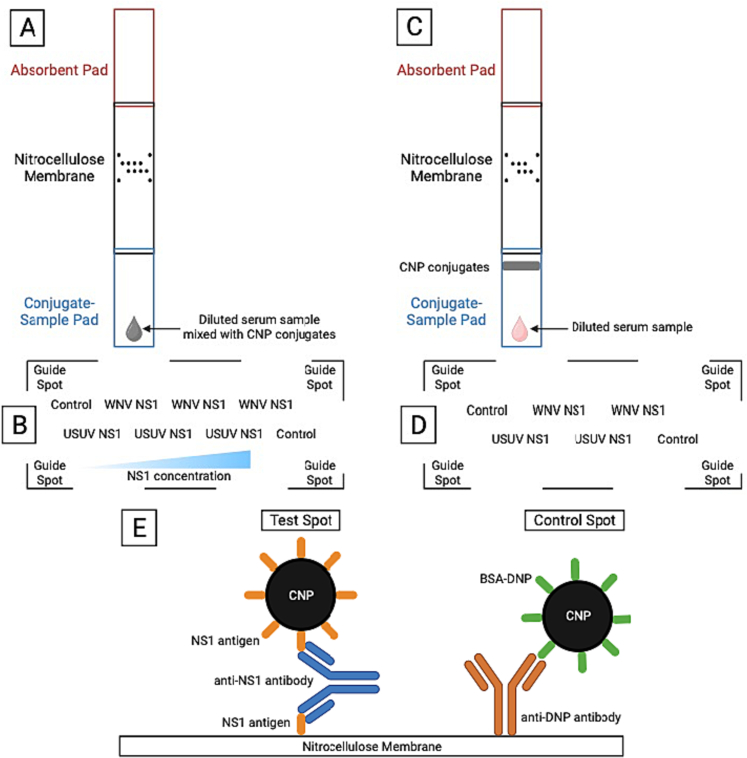
Overview of the LMIA formats used in this study and principle of double antigen approach. (A) Schematic of the LMIA for optimization. Diluted serum is mixed with detection antigen conjugates, pipetted onto conjugate-sample pad, and flows laterally towards the nitrocellulose membrane and absorbent pad. During flow, host antibodies specific for NS1 bind to NS1 detection antigen conjugates after which this complex is captured by NS1 capture antigen at the test spot, sandwiching the host antibody between two NS1 antigens (E). (B) Microarray layout of LMIA for optimization. WNV NS1 and USUV NS1 capture antigens increase in concentration from left to right (750, 1500, 3000 μg/mL), control spots are duplicates (2000 μg/mL), and guide spots are permanent spots to guide the reader towards the microarray. (C) Schematic of the optimized LMIA. Diluted serum is added onto conjugate-sample pad and flows laterally. At the CNP conjugate line, host antibodies specific for NS1 bind to NS1 detection antigen conjugates after which this complex is captured by NS1 capture antigen at the test spot (E). (D) Microarray layout of optimized LMIA. WNV NS1 and USUV NS1 capture antigens at test spots (3000 μg/mL) and control spots (2000 μg/mL) are respective duplicates. (E) Schematic representation of the double antigen approach sandwiching an anti-NS1 antibody between two NS1 antigens. At the test spot, a capture antigen captures the host antibody-detection antigen conjugate complex. At the control spot, an anti-DNP antibody captures a BSA-DNP conjugated CNP.

Certain LMIA parameters were optimized to detect WNV NS1 and USUV NS1-specific antibodies in serum from different species. Prior to spotting, WNV NS1 and USUV NS1 were concentrated using Amicon® Ultra-0.5 Centrifugal Filter Devices. Protein concentration was determined using a spectrophotometer (DeNovix DS-11 FX, Wilmington, USA). First, a 2.5 cm wide nitrocellulose membrane was placed onto a pressure sensitive adhesive backing card. Using a non-contact protein array spotter (sciFLEXARRAYER S3 spotter; Scienion, Berlin, Germany), 20 nL spots of WNV NS1 and USUV NS1 were spotted on the nitrocellulose membrane at concentrations of 750, 1500, and 3000 μg/mL (PBS, pH 8.8). 20 nL of guide (polymerInk VP240 blue non-soluble dye) and control spots (2000 μg/mL anti-DNP KLH) were also spotted as shown in Fig. 1B. Spotted nitrocellulose membranes were then allowed to dry at 37 °C for 1 h before a 2.1 cm absorbent pad was placed onto the backing card, overlapping the nitrocellulose membrane by 1 mm on the top end of the LMIA (Fig. 1A). A 2.2 cm wide sample/conjugate pad was prepared by first blocking in blocking buffer (BB, 100 mM, pH 8.8, containing 1% skim milk protein) and allowed to dry at 37 °C. This was then placed onto the backing card, overlapping the nitrocellulose membrane by 1 mm on the bottom end of the LMIA (Fig. 1A). Finally, 6 mm wide strips were cut using a programmable cutter (Kinematic Matrix 2360; Kinematic Automation Inc., Twain Harte, USA) and stored at room temperature with silica desiccants  (Multisorb Technologies, Inc., NY, USA) in aluminium pouches ( Nefab, Barneveld, The Netherlands) until further use.

### 2.5 Detection of antibodies using LMIAs for optimization

During preparation of LMIAs, blocking of non-specific binding was not used, therefore the assay running buffer (BB, 100 mM, 1% (*w/v*) skim milk protein, 0.05% (*v/v*) Tween20, pH 8.8) was sufficient for ‘blocking-on-the-run’. Three CNP formulations were prepared. Firstly, a CNP formulation of 2:1:1 WNV NS1 350 μg/mL CNP: USUV NS1 350 μg/mL CNP: BSA-DNP CNP 350 μg/mL. Secondly, a CNP formulation of 2:1:1 WNV NS1 175 μg/mL CNP: USUV NS1 175 μg/mL CNP: BSA-DNP CNP 350 μg/mL. Thirdly, a CNP formulation of 2:1:1 WNV NS1 88 μg/mL CNP: USUV NS1 88 μg/mL CNP: BSA-DNP CNP 350 μg/mL. Selected human, horse, and European jackdaw (*Corvus monedula*) sera were diluted 1:20 in assay running buffer and mixed with 1 μL of either the 350 μg/mL, 175 μg/mL, or 88 μg/mL formulation. Due to low whole blood volume, common blackbird (*Turdus merula*) serum was more difficult to separate from whole blood without haemolytic contaminants. This affected the background of the nitrocellulose membrane (not shown). It was therefore necessary to dilute *T. merula* serum samples 1:50 in assay running buffer. Data analysis of 1:20 or 1:50 dilutions were conducted using the same methods. However, it is acknowledged that a more diluted serum sample may lower the sensitivity of the LMIA presented. Prepared LMIA strips were placed into a plastic cassette which was then placed into a sciREADER LF1 real-time video reader (Scienion, Berlin, Germany). The diluted sample mixed with CNP formulation was then pipetted onto the LMIA sample/conjugate pad. Upon sample front detection, the sciREADER LF1 measured the spot intensity of the test and control spots after 30 min.

### 2.6 Preparation of optimized LMIAs

Aside from the test spots and the sample/conjugate pad, optimized LMIAs were prepared like LMIAs for optimization. For test spots, a non-contact protein array spotter was used to spot 20 nL of WNV NS1 and USUV NS1 at a concentration of 3000 μg/mL (PBS, pH 8.8) onto the nitrocellulose membrane (Fig. 1D). For the sample/conjugate pad, a CNP formulation of 2:1:1 WNV NS1 350 μg/mL CNP: USUV NS1 350 μg/mL CNP: BSA-DNP CNP 350 μg/mL was diluted in dilution buffer (BB,  100 mM, pH 8.8, 1% (*w/v*) skim milk protein, 3% (*w/v*) trehalose). This was sprayed onto a blocked sample/conjugate pad at 20 μL/cm using  a Biodot Dispense Platform ZX1010 (Biodot Inc., Irvine, CA, USA) before being allowed to dry at 37 °C and placed onto a backing card with a 2 mm overlap with the nitrocellulose membrane (Fig. 1C).

### 2.7 Detection of antibodies using optimized LMIA

Selected human, horse, *T. merula*, or *C. monedula* sera were diluted 1:20 in assay running buffer. *T. merula* sera were diluted 1:50 in assay running buffer. Prepared LMIA strips were placed into a plastic cassette which was then placed into a sciREADER LF1 real-time video reader. The diluted sample was then pipetted onto the LMIA sample/conjugate pad. Upon sample front detection, the sciREADER LF1 measured the spot intensity of the test and control spots after 30 min.

### 2.8 Detection of antibodies using protein microarray (PMA)

All serum samples were tested using the PMA method as previously described in detail with a few modifications [[20], [21], [22]]. 64 nitrocellulose glass slides (Sartorius, Gӧttingen, Germany) were spotted with USUV NS1 (The Native Antigen Company) and WNV NS1 (Sino Biological) in duplicate. Slides were blocked for 1 h using Blocker™ Blotto in TBS (Thermo Fischer Scientific, Waltham, MA, USA). For IgG or IgY antibody detection, slides were incubated for 1 h at 37 °C with a two-fold diluted human, horse, and *C. monedula* sera ranging from 1:20 to 1:2560. Due to low sample volume, *T. merula* sera were tested at one dilution (1:80). Between incubation steps, slides were washed with PBS 0.05% TWEEN® 20 (Sigma Aldrich). Subsequently, human sera were incubated for 1 h at 37 °C with a Cy5-fluorescent conjugated Fc-fragment specific IgG (Invitrogen, CA, USA); horse sera with an Alexa Fluor-647 fluorescent dye conjugated anti-equine Fc-fragment-specific IgG (Jackson ImmunoResearch, West Grove, USA); *C. monedula* and *T. merula* sera with Alexafluor-647 conjugated goat anti-duck IgY 647 (Jackson Immunoresearch Laboratories, Inc., West Grove, USA). Lastly, median fluorescence intensities were measured using a Tecan PowerScanner™ (Tecan Trading AG, Mannedorf, Switzerland). Fluorescent intensity of the spots was analysed using ImaGene 9.0 software (BioDiscovery inc. El Segundo, CA). For IgG, the fluorescent signals of the tested dilutions were used to calculate the half maximal effective concentration titres [23].

### 2.9 Detection of antibodies using focus reduction neutralization test (FRNT)

To confirm LMIA and PMA results, all sera were tested for the presence of neutralizing antibodies against WNV lineage 2 (B956, NCPV Porton Down #638, 2010) and USUV Africa-3 (*T. merula* NL isolate, 2016) by FRNT as previously described with some modifications [24].

Sera were heat-inactivated for 30 min at 56 *°C. Heat-inactivated sera were* 2-fold serially diluted, starting with 1:10. Subsequently, a virus suspension (800 plaque forming units (PFU) based on 24-h titrations) was added to a final concentration per well of 400 PFU and incubated for 1 h at 37 °C. Virus and serum mix was added to confluent monolayers of Vero cells (ATCC CCL-81) and incubated for 24 h at 37 °C. Cells were then fixed and permeabilized before staining for 1 h at 37 °C using polyclonal mouse anti-USUV NS1 antibody (1:10000, MyBioscource, MBS569354_1mg) or anti-WNV NS1 antibody (1:4000, IC12) (The Native Antigen Company, MAB12160–100). Following a PBS wash, secondary antibody staining was done for 1 h at 37 °C using goat anti-mouse IgG (H + L) cross-adsorbed horseradish peroxidase (HRP) (1:6000, Invitrogen, A16072). Following another PBS wash, TrueBlue Peroxidase Substrate (KPL TrueBlue, 5510–0030, Seracare) was added and plates were incubated in a dark at room temperature for 5–10 min. Following a last PBS wash, plates were air-dried and scanned by the CTL Immunospot scanner (S6 Ultimate-V Analyzer, CTL Analyzers LCC). FRNT titres were calculated based on a 90% reduction in infected cells counts (FRNT90). A reciprocal titre of ≥1:80 for WNV, ≥1:160 for USUV, and a ≥ 4-fold difference between WNV and USUV FRNT titres was deemed a positive result.

### 2.10 Data analysis

The sciREADER LF1 reader measures mean pixel intensity (i.e., the mean of all pixel intensities in the region of interest of a single test or control spot (ROI), referred to from here on as “spot intensity”). This measurement is expressed in arbitrary units (AU). The spot intensity data represents measurements that are compensated with respect to the background within the ROI; therefore, all presented spot intensities had been background corrected by the reader's software.

All subsequent data analysis was done using R software (version 4.2.3, packages: ggplot2, dplyr, and tidyverse) or GraphPad Prism (version 9.3.1 (471)). For each LMIA, individual test spots were corrected with respect to the control spots on the same LMIA. For LMIAs for optimization, this was done by dividing the spot intensity of a single test spot by the mean of the spot intensities of the duplicate control spots. For optimized LMIAs, this was done by dividing the mean of the spot intensities of the duplicate test spots by the mean of the spot intensities of the duplicate control spots.

Figures for LMIA optimization were created using R software and figures for optimized LMIA were created using R software and GraphPad Prism. For optimized LMIAs, a Wilcoxon signed-rank test was used to determine the significance of difference between homologous and heterologous binding events (e.g., anti-WNV NS1 antibodies binding WNV NS1 compared to anti-WNV NS1 antibodies binding USUV NS1) (*p* < 0.05). Furthermore, for each species tested using the optimized LMIA, mean fold differences between WNV NS1 and USUV NS1 spot intensities were calculated. For FRNT90-confirmed WNV positive human, horse, and *C. monedula* sera this was calculated by dividing the mean spot intensity of WNV NS1 by mean spot intensity for USUV NS1. For FRNT90-confirmed USUV positive *T. merula* sera, this was calculated by dividing the mean spot intensity of USUV NS1 by the mean spot intensity of WNV NS1. Using GraphPad Prism, a receiver operating characteristic (ROC) curve was generated comparing WNV NS1 LMIA and WNV FRNT90-confirmed horse, human, and *C. monedula* sera. GraphPad Prism was also used to determine the area under the ROC curve (AUC) and to determine the optimal sensitivity and specificity of WNV NS1 LMIA when compared to WNV FRNT90 results. A ROC curve was not generated comparing USUV NS1 LMIA and USUV FRNT90-confirmed *T. merula* sera because the sample size was too small.

## 3 Results

### 3.1 Optimization of LMIAs with respect to sensitivity and specificity

Prior to testing of a larger serum panel, two LMIA parameters were optimized with respect to sensitivity and specificity. The two parameters were 1) the amount of WNV NS1 or USUV NS1 detection antigen conjugated to CNPs and 2) the concentration of WNV NS1 or USUV NS1 capture antigen spotted onto the nitrocellulose membrane.

#### 3.1.1 Optimizing the amount of detection antigen conjugated to CNP

**Fig. 2:**
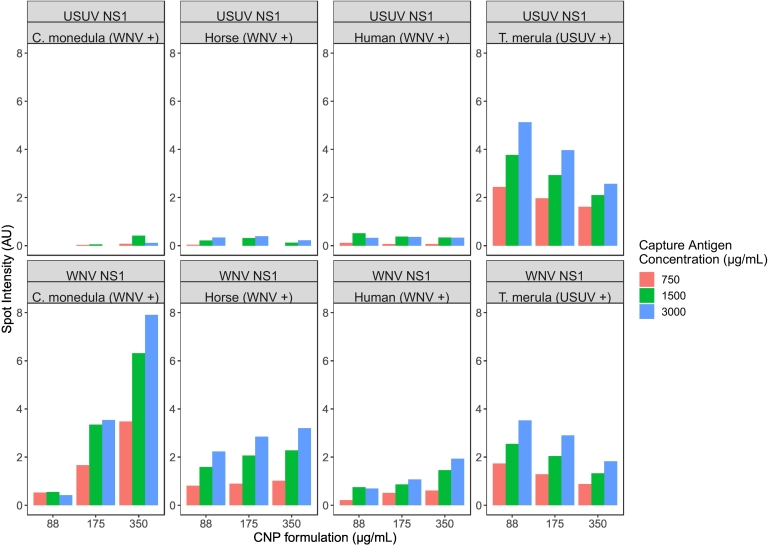
Optimizing the amount of detection antigen conjugated to CNP and the concentration of capture antigen on nitrocellulose membrane across four species. 20 nL of WNV NS1 and USUV NS1 were spotted onto the nitrocellulose membrane at 750, 1500, and 3000 μg/mL (orange, green, and blue bars, respectively). Above each graph, capture antigen measured and FRNT90-confirmed positive serum are indicated. 88, 175, or 350 μg/mL of detection antigen were conjugated to CNP (left to right, following identical bar colour). Bar graphs depict spot intensity of each spot as measured by the sciREADER LF1 after 30 min.

To optimize the amount of WNV NS1 or USUV NS1 antigen conjugated to CNPs, the amount of each antigen was decreased two-fold from 350 to 88 μg detection antigen/mL 0.2% CNP suspension. The effect on spot intensity as measured by the sciREADER LF1 was observed across four species (Fig. 2).

Aside from the *T. merula* sample, an increase in the amount of detection antigen conjugated to CNP correlated with an increase in spot intensity of a homologous binding event. Considering the findings that across three of the four species tested (horse, human, and *C. monedula*) the spot intensity was highest for 350 μg/mL detection antigen conjugate, this was determined to be the optimal amount of detection antigen conjugated to CNP.

#### 3.1.2 Optimizing the concentration of capture antigen spotted onto nitrocellulose membrane

To optimize the concentration of capture antigen spotted onto nitrocellulose membrane, the concentration of WNV NS1 or USUV NS1 antigen spotted onto the nitrocellulose membrane was increased in two-fold steps from 750 μg/mL to 3000 μg/mL. The effect on spot intensity as measured by the sciREADER LF1 was observed across four different species (Fig. 2).

Across the four different species, an increase in the concentration of capture antigen correlated to an increase in spot intensity of a homologous binding event. Across the four species tested, this correlation also applied to the three different CNP formulations of 350, 175, and 88 μg/mL detection antigen conjugates. Considering the findings that across all species and detection antigen conjugate formulations the spot intensity is highest for 3000 μg/mL spots, this was determined to be the optimum capture antigen concentration to continue with.

#### 3.1.3 Chosen CNP parameters do not show visible background spot development

After the parameters were optimized, it was important to verify that FRNT90-confirmed negative sera from different species did not develop a visible background spot. In parallel to the above experiments, FRNT90-confirmed WNV and USUV negative sera from human, *C. monedula*, and horse were tested.

For each parameter optimized (capture antigen concentration and amount of detection antigen conjugated to CNP) no visible background spot development is observed by eye (Supp. Fig. 1). This applies to the three FRNT90-confirmed negative species tested. This indicates that one can be confident in the spot development and intensity observed by the sciREADER LF1 in confirmed WNV or USUV positive and negative sera.

### 3.2 Optimized LMIA sensitivity and specificity across species

**Fig. 3:**
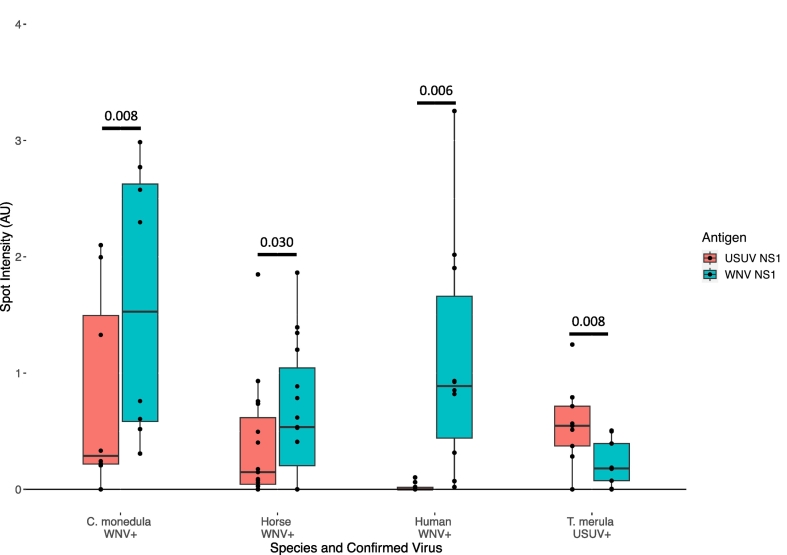
Performance of the optimized LMIA across four species. Box plots depicting USUV NS1 and WNV NS1 spot intensities as measured by the sciREADER LF1 after 30 min for *C. monedula* (*n* = 8), horse (*n* = 15), human (*n* = 11), and *T. merula* (*n* = 10). For each individual sample tested, spot intensities are shown as black dots. Comparison of WNV NS1 and USUV NS1 spot intensities for each species (Wilcoxon signed-rank test).

With the two parameters chosen, optimized LMIAs were used to test sera from FRNT90-confirmed *T. merula*, horses, humans, and *C. monedula*. Across the four species, the median spot intensity for a homologous binding event is higher than it is for a heterologous binding event (Fig. 3). For USUV positive *T. merula*, the median spot intensity of USUV NS1 and WNV NS1 are 0.55 and 0.18, respectively. For WNV positive horses, the median spot intensity of USUV NS1 and WNV NS1 are 0.15 and 0.54, respectively. For WNV positive humans, the median spot intensity of USUV NS1 and WNV NS1 are, 0.00 and 0.88, respectively. For WNV positive *C. monedula*, the median spot intensity of USUV NS1 and WNV NS1 are, 0.29 and 1.53, respectively.

However, cross reactivity for non-human species appeared to be higher than for humans. In human sera tested, there is a mean fold-difference in spot intensity of 3.38. For *C. monedula*, horse, and *T. merula* sera tested the mean fold-differences are 2.45, 2.11, and 2.29, respectively. However, significant differences were observed between USUV NS1 and WNV NS1 spot intensities for tested *T. merula*, horse, human, and *C. monedula* sera (Fig. 3) (Wilcoxon signed-rank test; *p* = 0.008, 0.030, 0.006, and 0.008, respectively).

**Fig. 4:**
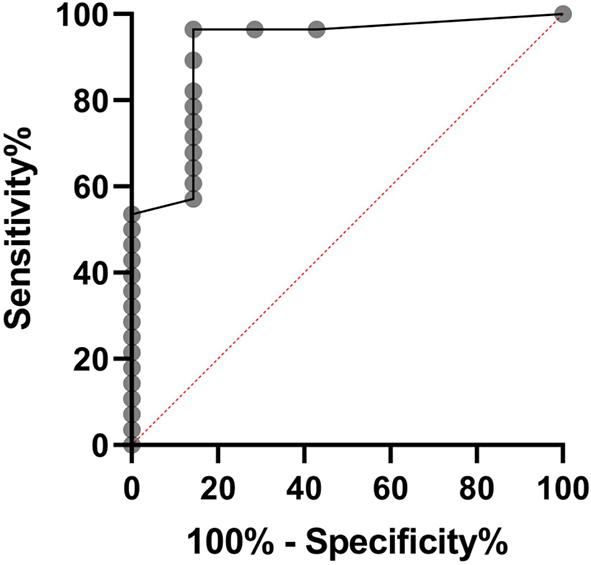
ROC curve comparing WNV NS1 LMIA to WNV FRNT90 in horse, human, and *C. monedula* sera. ROC curve of LMIA WNV NS1 spot intensity relative to corresponding “gold standard” WNV FRNT90-confirmed horse, human, and *C. monedula* sera (n = 11, 15, and 8, respectively; *n* = 34 total; AUC = 0.916). An AUC of 0.916 indicates a good correlation between WNV NS1 LMIA and current “gold standard” results. Each grey point represents a sensitivity/specificity pair corresponding to a decision threshold. The most optimal derived sensitivity and specificity were determined to be 96% and 86%, respectively.

Using a ROC curve, LMIA sensitivity and specificity for WNV NS1-specific antibodies in human (*n* = 11), horse (n = 15), and *C. monedula* (n = 8) sera were collectively compared to WNV FRNT90 results (Fig. 4). The area under the ROC curve (AUC = 0.916) indicates a good correlation between WNV NS1 LMIA and “gold standard” WNV FRNT90-confirmed results. The most optimal derived sensitivity and specificity of the WNV NS1 LMIA relative to corresponding FRNT90-confirmed sera were determined to be 96% and 86%, respectively. LMIA USUV NS1 results for tested *T. merula* sera were compared to USUV FRNT90 results (not shown). Of the 10 *T. merula* samples tested, 9 were positive and 1 negative on both the USUV NS1 LMIA and the USUV FRNT90.

FRNT90, LMIA, and PMA results for each individual serum tested are presented in more detail (Supp. Table 1).

## 4 Discussion

The aim of this study was to develop a species-independent LMIA able to detect and distinguish between WNV NS1 and USUV NS1-specific antibodies. Utilizing a double antigen approach, the ability of the optimized LMIA to detect and distinguish between those antibodies in human, horse, European jackdaw (*C. monedula*), and common blackbird (*T. merula*) sera was demonstrated. Firstly, two parameters were optimized, the concentration of capture antigen and the amount of detection antigen conjugated to CNPs. Following optimization, a larger panel of sera were tested on the optimized LMIA, and results were compared to FRNT90.

As hypothesized, a higher capture antigen concentration correlated with a higher spot intensity. However, the hypothesis that an increasing amount of detection antigen conjugated to CNP correlates to higher spot intensity was only true for three of the four species tested. In *T. merula* the opposite was observed. Nonetheless, this reverse correlation was reproducible across multiple, independent *T. merula* samples (not shown). As stated earlier, the primary difference observed between *T. merula* and other sera tested was the quality of the sera. Tested *T. merula* sera came from live *T. merula*, small passerine birds from which collecting sufficient volumes of whole blood without harming the birds can be difficult [20]. Separating serum/plasma from whole blood was therefore difficult and may have led to haemolytic *T. merula* sera containing haemoglobin and other erythrocytic components. While a literature search did not yield any results indicating a positive or negative correlation between avian haemolytic serum and spot intensity, the observations in this study indicate that avian haemolytic serum may indeed influence spot intensity.

It is also acknowledged that further optimizing both parameters may be necessary. It has been suggested that capture antigen concentration should be maximized in the analytical zone without exceeding the sorption capacity of the nitrocellulose membrane [25]. However, financial and experimental restraints did not allow further concentration of the capture antigens used in this study. It is also important to note that the unit cost of the assay increases with an increase in the concentration of the assay's capture antigen; a point of consideration when deploying such an assay in resource poor regions. With respect to conjugation of detection antigen to CNP, the stability of the CNP suspension is maximized at 350 μg/mL in 0.2% (*w/v*) CNP suspension [26]. Furthermore, even if it was possible to conjugate more detection antigen to CNP, the number of functional detection antigens (i.e., detection antigen epitopes available for binding) would become saturated [27].

Optimized LMIAs were used to test *T. merula*, horse, human, and *C. monedula* sera. As hypothesized, varying degrees of cross-reactivity were observed. While for each species there was a significant difference observed between a homologous and heterologous binding event, the degree of cross-reactivity differed between human and non-human species.

For *T. merula* and *C. monedula* this could be due to avian species having a narrower repertoire of immune genes than mammals, leading to a reduced ability to detect and distinguish between certain viruses [28]. For horses, the higher cross-reactivity observed may be attributed to the higher viscosity of horse blood when compared to humans. During flow through the nitrocellulose fibres of the LMIA, a higher viscosity sample may increase non-specific binding events [21]. As stated earlier, WNV and USUV are often found to co-circulate in the same region, and it may well be true that both WNV and USUV antibodies were present in the serum samples tested. It is acknowledged that LMIA results must still be confirmed and validated using gold-standard, laboratory-based assays such as NTs. To reiterate, the presented LMIA is not meant to replace well established serological assays such as NTs, but rather serve as an on-site screening tool in surveillance and monitoring.

Limitations of this study were partially attributable to the volume of serum samples tested and the number of serum samples available to test. While the outcomes of this study are promising, further optimization should be conducted to determine the optimal dilution ratio of serum samples to be tested. However, due to low sample volumes this was not possible at the time. Furthermore, a larger panel of serum samples would allow for more robust statistics to be performed, something also not available at the time. Nevertheless, building on the presented proof of concept could lead to the development of a useful tool to be used in the frontline surveillance of WNV and USUV in many affected parts of the globe. For example, the presented LMIA may be used as an on-site tool to quickly screen samples collected in a remote region with no readily available access to a laboratory. An initial determination of seropositivity or negativity would then allow a frontline decision to be taken as to whether a sample should or should not be sent to a dedicated laboratory for further testing, thus saving time and resources.

## 5 Conclusions

The feasibility of developing a species-independent LMIA able to detect and distinguish between WNV NS1 and USUV NS1-specific antibodies was demonstrated. An LMIA like the one presented would be a useful screening tool for the on-site analysis of WNV and USUV in diverse regions of the world. Further developing this tool to incorporate WNV and USUV E antigen would also prove useful as E antigens are the target of neutralizing antibodies [29]. This could be important as it would allow LMIA results to be compared to both PMA (NS1-based) and NTs (E-based). Lastly, a fieldable assay should be able to test not only serum samples, but also whole blood and tissue samples. Specialized membranes exist that capture blood cells and allow serum/plasma to migrate across the LMIA.

## Authors contributions

BG prepared all LMIAs used in this study, tested all the sera presented on LMIA, analysed LMIA data, and wrote the first draft of the manuscript. FC and AvdL tested samples on, analysed results of, and wrote methods of PMA and FRNT90. FC, AvdL, and EdB provided technical assistance with respect to PMA and FRNT90. AvA, RS, EV, EdB, AG, FC, and AvdL contributed to reviewing and editing. All authors read and made significant inputs and agreed on the last version of the paper.

## Funding

This publication is part of the project ‘Preparing for Vector-Borne Virus Outbreaks in a Changing World: A One Health Approach’ (NWA.1160.1S.210), which is (partly) financed by the Dutch Research Council (NWO)).

## Declaration of Competing Interest

None to declare.

## Data Availability

Data not already included in supplementary materials will be made available upon request.

## Appendix A Supplementary data

Supp. Fig. 1 Chosen LMIA parameters do not show visible background spot development. Image 1

Supplementary Table 1 Summary of LMIA, PMA, and FRNT90 results for each serum tested. Image 2
