# Supplementary material for: A species-independent lateral flow microarray immunoassay to detect WNV and USUV NS1-specific antibodies in serum
Source: One Health. 2023 Dec 27;18:100668. doi: 10.1016/j.onehlt.2023.100668 (PMC10796932; doi:10.1016/j.onehlt.2023.100668)
Supplement: Supplementary file 1 — Supp. Fig. 1 Chosen LMIA parameters do not show visible background spot development. [file mmc1.docx]

**Supplementary figures and tables**

|  | Detection antigen conjugated to CNP (µg/mL) | | |
| --- | --- | --- | --- |
|  | 88 | 175 | 350 |
| Human negative | 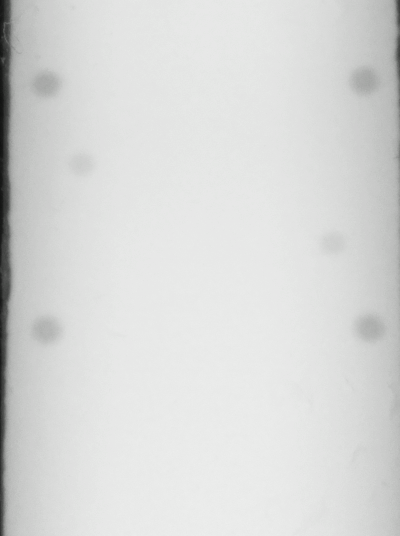 | 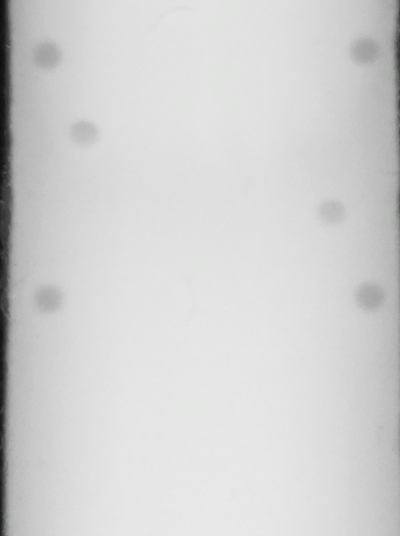 | 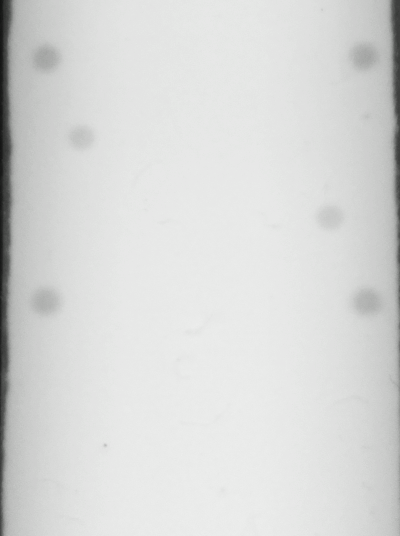 |
| Horse negative | 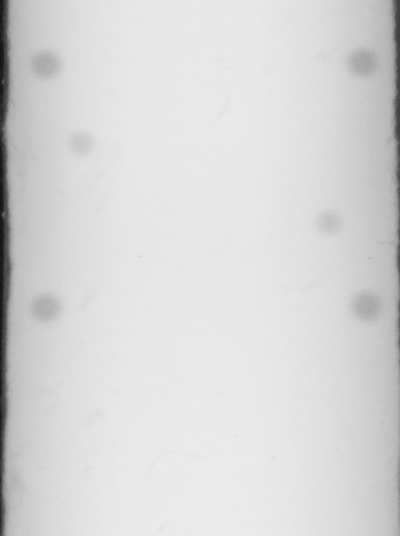 | 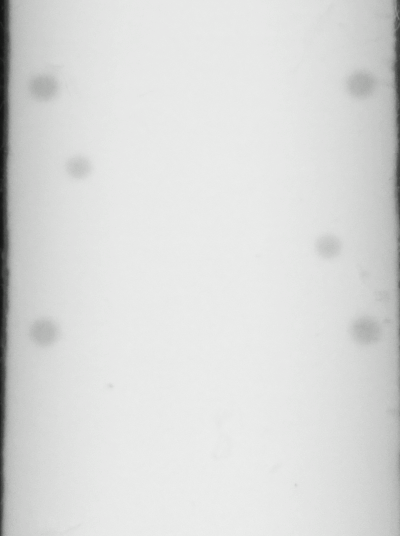 | 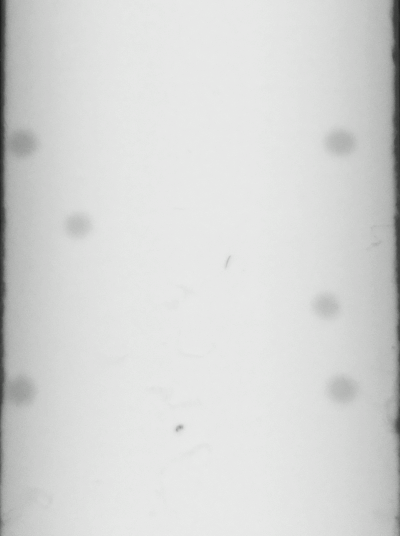 |
| *T. merula* negative | 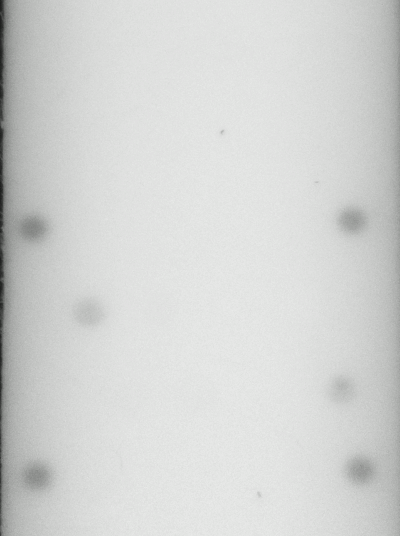 | 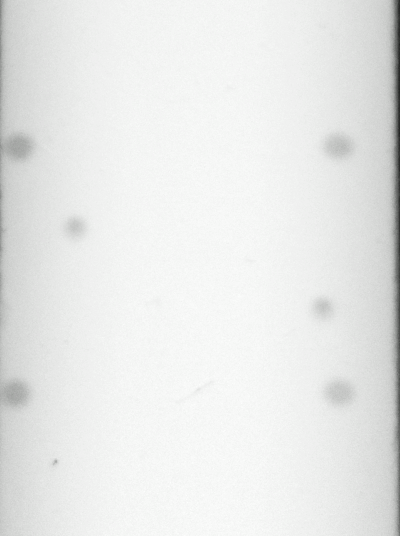 | 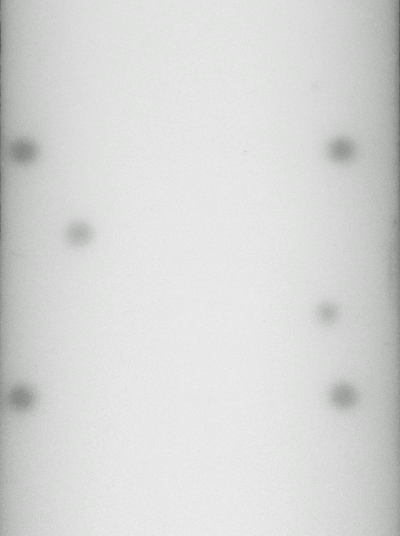 |

**Supp. Fig. 1** Chosen LMIA parameters do not show visible background spot development.

20 nL of WNV NS1 and USUV NS1 were spotted onto the nitrocellulose membrane at 750, 1500, and 3000 µg/mL. 20 nL of anti-DNP antibody were spotted in duplicate at 2000 µg/mL. 88, 175, or 350 µg/mL of detection antigen were conjugated to CNP. Image taken by the sciREADER LF1 after 30 minutes show that only control spots, and not test spots, develop.
