# Supplementary material for: A species-independent lateral flow microarray immunoassay to detect WNV and USUV NS1-specific antibodies in serum
Source: One Health. 2023 Dec 27;18:100668. doi: 10.1016/j.onehlt.2023.100668 (PMC10796932; doi:10.1016/j.onehlt.2023.100668)
Supplement: Supplementary file 2 — Supplementary Table 1 Summary of LMIA, PMA, and FRNT90 results for each serum tested. [file mmc2.docx]

**Supplementary Table 1** Summary of LMIA, PMA, and FRNT90 results for each serum tested.

| **Species** | **ID** | **LMIA**  **WNV NS1** | **LMIA**  **USUV NS1** | **PMA**  **WNV NS1 (cut-off > 20)** | **PMA**  **USUV NS1 (cut-off > 20)** | **FRNT90**  **WNV (cut-off ≥80)** | **FRNT90**  **USUV (cut-off ≥160)** |
| --- | --- | --- | --- | --- | --- | --- | --- |
| Human | 1 | + (0.82) | - (0.00) | 1048 | 40 | 10 | 10 |
|  | 2 | + (0.31) | - (0.00) | 1129 | NA | 640 | 10 |
|  | 3 | + (0.93) | - (0.00) | 1167 | 44 | 1280 | 10 |
|  | 4 | + (0.92) | - (0.02) | 1032 | NA | 640 | 10 |
|  | 5 | + (3.25) | - (0.00) | 954 | <20 | 1280 | 10 |
|  | 6 | + (1.90) | - (0.00) | <20 | <20 | 320 | 10 |
|  | 7 | + (0.85) | + (0.06) | >1280 | 101 | 2560 | 10 |
|  | 8 | - (0.07) | - (0.00) | 235 | <20 | 10 | 10 |
|  | 9 | - (0.02) | - (0.00) | 353 | <20 | 10 | 10 |
|  | 10 | + (2.02) | + (0.10) | 611 | 28 | 2560 | 20 |
|  | 11 | - (0.00) | - (0.00) | 20 | 20 | 10 | 10 |
| Horse | 1 | + (1.20) | + (1.85) | 811 | 898 | 2560 | 40 |
|  | 2 | - (0.00) | - (0.00) | 223 | 20 | 320 | 10 |
|  | 3 | + (0.79) | - (0.09) | >1280 | 268 | 1280 | 10 |
|  | 4 | + (0.54) | - (0.03) | 1067 | 230 | 1280 | 10 |
|  | 5 | + (0.62) | - (0.09) | >1280 | 249 | 1280 | 20 |
|  | 6 | + (0.41) | + (0.15) | 1067 | 207 | 2560 | 10 |
|  | 7 | + (0.53) | + (0.17) | 1018 | 125 | 2560 | 10 |
|  | 8 | + (1.86) | + (0.93) | >1280 | 233 | 2560 | 10 |
|  | 9 | + (0.41) | + (0.76) | 994 | 217 | 1280 | 10 |
|  | 10 | - (0.00) | + (0.06) | 80 | 52 | 1280 | 20 |
|  | 11 | + (1.39) | + (0.50) | >1280 | 274 | 2560 | 10 |
|  | 12 | - (0.00) | - (0.00) | 20 | 20 | 10 | 10 |
|  | 13 | - (0.00) | - (0.00) | 20 | 20 | 1280 | 10 |
|  | 14 | + (1.35) | + (0.74) | 250 | 20 | 2560 | 10 |
|  | 15 | + (0.89) | + (0.40) | 307 | 20 | 2560 | 10 |
| *C. monedula* | 1 | + (0.52) | + (0.24) | 78 | 20 | 1280 | 80 |
|  | 2 | + (2.30) | + (1.33) | 57 | 20 | 2560 | 40 |
|  | 3 | + (2.58) | + (2.10) | 229 | 20 | 2560 | 320 |
|  | 4 | + (0.76) | + (0.33) | 72 | 20 | 2560 | 80 |
|  | 5 | + (3.00) | + (2.00) | 346 | 20 | 2560 | 80 |
|  | 6 | + (0.61) | + (0.22) | 65 | 20 | 2560 | 80 |
|  | 7 | + (2.77) | + (0.21) | 419 | 34 | 2560 | 80 |
|  | 8 | + (0.31) | - (0.00) | 116 | 20 | 2560 | 80 |
|  |  |  |  | **Fluorescence (cut-off > 20000)** | |  |  |
| *T. merula* | 1 | + (0.07) | + (0.57) | 57137 | 61286 | 10 | 1280 |
|  | 2 | - (0.00) | + (0.51) | 60818 | 61130 | 10 | 1280 |
|  | 3 | + (0.19) | + (0.55) | 54500 | 60545 | 320 | 320 |
|  | 4 | + (0.50) | + (1.25) | 58084 | 60126 | 40 | 1280 |
|  | 5 | + (0.40) | + (0.71) | 61751 | 62338 | 80 | 640 |
|  | 6 | - (0.00) | - (0.00) | 56469 | 22954 | 10 | 10 |
|  | 7 | + (0.18) | + (0.28) | 60736 | 61033 | 320 | 1280 |
|  | 8 | + (0.18) | + (0.37) | 60496 | 60982 | 10 | 320 |
|  | 9 | + (0.50) | + (0.80) | 60584 | 61008 | 10 | 320 |
|  | 10 | + (0.07) | + (0.57) | 57137 | 61286 | 10 | 1280 |
| Total | 44 |  |  |  | |  |  |

LMIA WNV NS1 and USUV NS1 spot intensity is shown in parentheses with + and – indicating, respectively, a spot development or lack thereof as observed by eye. WNV and USUV FRNT90 and WNV NS1 and USUV NS1 PMA cut-offs indicate if sample is positive or negative for the virus indicated. Due to low sample volume for *T. merula* samples, PMA tests were only conducted at a dilution of 1:80 and results are displayed as fluorescence with cut-off of 20000.
